# *catena*-Poly[[dichloridozinc(II)]-μ-[1,1′-(butane-1,4-di­yl)diimidazole-κ^2^ *N* ^3^:*N* ^3′^]]

**DOI:** 10.1107/S1600536810046611

**Published:** 2010-11-17

**Authors:** Jing Lin, Wen-Lian Cai, Xi-Zhong Li, Sen-Ke Huang

**Affiliations:** a Department of Pharmacy, Xiamen University, Xiamen, Fujian 363105, People’s Republic of China; b Department of Chemistry and Environmental Science, Zhangzhou Normal University, Zhangzhou, Fujian,363000, People’s Republic of China

## Abstract

The title one-dimensional coordination polymer, [ZnCl_2_(C_10_H_14_N_4_)]_*n*_, was synthesized by hydro­thermal methods from ZnCl_2_ and 1,1′-(butane-1,4-di­yl)diimidazole. The Zn atom is coordinated by two chloride ions and two N atoms from two symmetry-independent organic ligands and shows a distorted tetra­hedral coordination geometry. The 1,1′-(butane-1,4-di­yl)diimidazole ligands are located around two sets of inversion centers and bridge Zn^II^ ions, forming a zigzag polymeric chain. C—H⋯Cl hydrogen bonding results in the formation of a three-dimensional supra­molecular network

## Related literature

For general background to this work, see: Hamada *et al.* (2004 ▶); Wang *et al.* (2006 ▶).
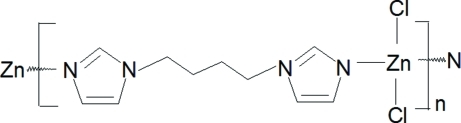


## Experimental

### 

#### Crystal data

[ZnCl_2_(C_10_H_14_N_4_)]
*M* *_r_* = 326.52
Monoclinic, 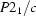

*a* = 7.8583 (16) Å
*b* = 11.689 (2) Å
*c* = 15.882 (3) Å
β = 93.82 (3)°
*V* = 1455.6 (5) Å^3^
*Z* = 4
Mo *K*α radiation
μ = 2.04 mm^−1^
*T* = 293 K
0.34 × 0.27 × 0.22 mm

#### Data collection

Siemens SMART CCD area-detector diffractometer
Absorption correction: multi-scan (*SADABS*; Sheldrick, 1996 ▶) *T*_min_ = 0.428, *T*_max_ = 0.731
13865 measured reflections
3309 independent reflections
2701 reflections with *I* > 2σ(*I*)
*R*_int_ = 0.036

#### Refinement

*R*[*F* ^2^ > 2σ(*F* ^2^)] = 0.039
*wR*(*F* ^2^) = 0.126
*S* = 1.01
3309 reflections
154 parameters
H-atom parameters constrained
Δρ_max_ = 1.33 e Å^−3^
Δρ_min_ = −0.35 e Å^−3^

### 

Data collection: *SMART* (Siemens, 1994 ▶); cell refinement: *SAINT* (Siemens, 1994 ▶); data reduction: *SAINT*; program(s) used to solve structure: *SHELXS97* (Sheldrick, 2008 ▶); program(s) used to refine structure: *SHELXL97* (Sheldrick, 2008 ▶); molecular graphics: *SHELXTL* (Sheldrick, 2008 ▶); software used to prepare material for publication: *SHELXTL*.

## Supplementary Material

Crystal structure: contains datablocks I, global. DOI: 10.1107/S1600536810046611/gk2314sup1.cif

Structure factors: contains datablocks I. DOI: 10.1107/S1600536810046611/gk2314Isup2.hkl

Additional supplementary materials:  crystallographic information; 3D view; checkCIF report

## Figures and Tables

**Table 1:** Hydrogen-bond geometry (Å, °)

*D*—H⋯*A*	*D*—H	H⋯*A*	*D*⋯*A*	*D*—H⋯*A*
C3—H3*A*⋯Cl2^i^	0.93	2.77	3.601 (4)	149
C6—H6*A*⋯Cl1^ii^	0.93	2.65	3.553 (3)	164

Symmetry codes: (i) 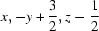; (ii) 
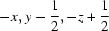
.

**Fig. 1.**
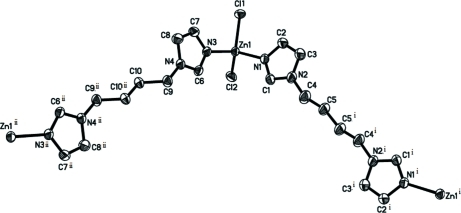
View of the title coordination polymer showing the atom-labelling scheme. Displacement ellipsoids are drawn at the 35% probability level. Symmetry code: (i) -x, -1/2 + y, 1/2 - z; (ii) x, 3/2 - y, -1/2 + z.

**Fig. 2.**
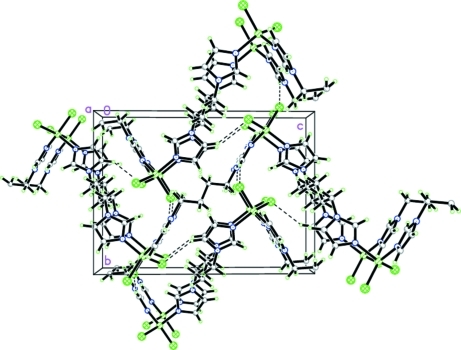
Crystal packing viewed along the a axis. C-H···Cl contacts are shown with dashed lines

## supplementary crystallographic information

### Comment

The chemistry of novel metal–organic coordination complexes has attached more and more attention in recent years because of their interesting topologies and unexpected properties for potential applications . Recently, there has been increasing interest in zinc–halogen compounds because of their applications in molecular materials (Hamada *et al.* 2004; Wang *et al.*, 2006). In this communication, we have introduced 1,1'-(butane-1,4-diyl)diimidazole (bbi) as a bridging ligand which favors crystal growth of the 1-D chain-like polymer. Through a mild-temperature hydrothermal process, we have successfully synthesized the title crystalline Cl-coordinated Zn complex, [ZnCl_2_(C_10_H_14_N_4_)]_n_, (I).

The molecular structure of (I) is shown in Fig. 1. The compound features 1-D chain-like polymer complex, in which the Zn atom is coordinated by two Cl anions and two N atoms from two bbi ligands in a distorted tetrahedral geometry, in which the Zn—Cl (2.238 (1) and 2.2567 (9) Å) and Zn—N(2.010 (2) and 2.016 (3) Å) bond lengths are in the expected ranges. Each bbi ligand in the title compound is located on an inversion center and bridges Zn^II^ ions, forming a zigzag polymeric chain with the adjacent Zn···Zn separation of 13.971 Å.

The strong C—H···Cl hydrogen bonding results in the formation of a 3-D supramolecular network, as shown in Fig. 2.

### Experimental

The hydrothermal reaction of ZnCl_2 _(0.041 g, 0.3 mmol), bbi (0.076 g, 0.4 mmol) and water (15.0 ml) was carried out at 423 K for 3 d. After cooling to room temperature at a rate of 5 K h^-1^, block-shaped colorless crystals of the title compound suitable for X-ray analysis were obtained.

### Refinement

The C-bound H atoms were positioned geometrically, with C—H = 0.93 - 0.97 Å and all refined as riding with *U*_iso_(H) = 1.2*U*_eq_(C). The crystal exhibited minor twinning which was not accounted for.

### Crystal data

**Table:**

[ZnCl_2_(C_10_H_14_N_4_)]	*F*(000) = 664
*M**_r_* = 326.52	*D*_x_ = 1.490 Mg m^−^^3^
Monoclinic, *P*2_1_/*c*	Mo *K*α radiation, λ = 0.71073 Å
Hall symbol: -P 2ybc	Cell parameters from 13865 reflections
*a* = 7.8583 (16) Å	θ = 3.1–27.4°
*b* = 11.689 (2) Å	µ = 2.04 mm^−^^1^
*c* = 15.882 (3) Å	*T* = 293 K
β = 93.82 (3)°	Block, colorless
*V* = 1455.6 (5) Å^3^	0.34 × 0.27 × 0.22 mm
*Z* = 4	

### Data collection

**Table:**

Siemens SMART CCD area-detector diffractometer	3309 independent reflections
Radiation source: fine-focus sealed tube	2701 reflections with *I* > 2σ(*I*)
graphite	*R*_int_ = 0.036
Detector resolution: 0 pixels mm^-1^	θ_max_ = 27.4°, θ_min_ = 3.1°
φ and ω scans	*h* = −10→8
Absorption correction: multi-scan (*SADABS*; Sheldrick, 1996)	*k* = −15→15
*T*_min_ = 0.428, *T*_max_ = 0.731	*l* = −20→20
13865 measured reflections	

### Refinement

**Table:**

Refinement on *F*^2^	Primary atom site location: structure-invariant direct methods
Least-squares matrix: full	Secondary atom site location: difference Fourier map
*R*[*F*^2^ > 2σ(*F*^2^)] = 0.039	Hydrogen site location: inferred from neighbouring sites
*wR*(*F*^2^) = 0.126	H-atom parameters constrained
*S* = 1.01	*w* = 1/[σ^2^(*F*_o_^2^) + (0.0822*P*)^2^ + 0.5697*P*] where *P* = (*F*_o_^2^ + 2*F*_c_^2^)/3
3309 reflections	(Δ/σ)_max_ = 0.001
154 parameters	Δρ_max_ = 1.33 e Å^−^^3^
0 restraints	Δρ_min_ = −0.35 e Å^−^^3^

### Special details

**Table:**

Geometry. All e.s.d.'s (except the e.s.d. in the dihedral angle between two l.s. planes) are estimated using the full covariance matrix. The cell e.s.d.'s are taken into account individually in the estimation of e.s.d.'s in distances, angles and torsion angles; correlations between e.s.d.'s in cell parameters are only used when they are defined by crystal symmetry. An approximate (isotropic) treatment of cell e.s.d.'s is used for estimating e.s.d.'s involving l.s. planes.
Refinement. Refinement of *F*^2^ against ALL reflections. The weighted *R*-factor *wR* and goodness of fit *S* are based on *F*^2^, conventional *R*-factors *R* are based on *F*, with *F* set to zero for negative *F*^2^. The threshold expression of *F*^2^ > σ(*F*^2^) is used only for calculating *R*-factors(gt) *etc*. and is not relevant to the choice of reflections for refinement. *R*-factors based on *F*^2^ are statistically about twice as large as those based on *F*, and *R*- factors based on ALL data will be even larger.

### Fractional atomic coordinates and isotropic or equivalent isotropic displacement parameters (Å^2^)

**Table:**

	*x*	*y*	*z*	*U*_iso_*/*U*_eq_	
Zn1	0.03756 (4)	0.88757 (3)	0.221856 (19)	0.03812 (15)	
Cl1	0.16460 (10)	1.03238 (7)	0.15605 (5)	0.0490 (2)	
Cl2	−0.16703 (11)	0.94302 (9)	0.30450 (5)	0.0595 (3)	
N1	−0.0583 (3)	0.7849 (2)	0.12834 (15)	0.0459 (6)	
N2	−0.1741 (4)	0.6401 (3)	0.05838 (18)	0.0564 (7)	
N3	0.2244 (3)	0.7996 (2)	0.28566 (15)	0.0414 (5)	
N4	0.3570 (3)	0.6570 (2)	0.35199 (15)	0.0418 (5)	
C1	−0.1396 (5)	0.6874 (3)	0.1340 (2)	0.0561 (9)	
H1A	−0.1695	0.6554	0.1845	0.067*	
C2	−0.0383 (5)	0.7996 (3)	0.0444 (2)	0.0604 (9)	
H2A	0.0175	0.8607	0.0209	0.072*	
C3	−0.1123 (6)	0.7112 (4)	0.0007 (2)	0.0692 (11)	
H3A	−0.1192	0.7015	−0.0575	0.083*	
C4	−0.2665 (6)	0.5326 (3)	0.0393 (3)	0.0721 (11)	
H4A	−0.2590	0.4840	0.0890	0.087*	
H4B	−0.2132	0.4926	−0.0055	0.087*	
C5	−0.4511 (6)	0.5543 (3)	0.0128 (3)	0.0687 (11)	
H5A	−0.4577	0.6072	−0.0344	0.082*	
H5B	−0.5053	0.5904	0.0591	0.082*	
C6	0.2058 (4)	0.7031 (3)	0.32738 (19)	0.0443 (7)	
H6A	0.1011	0.6712	0.3383	0.053*	
C7	0.3969 (4)	0.8154 (3)	0.2830 (2)	0.0539 (8)	
H7A	0.4483	0.8769	0.2576	0.065*	
C8	0.4809 (4)	0.7279 (3)	0.3232 (2)	0.0565 (9)	
H8A	0.5984	0.7176	0.3299	0.068*	
C9	0.3835 (4)	0.5474 (3)	0.3955 (2)	0.0487 (7)	
H9A	0.4469	0.4969	0.3607	0.058*	
H9B	0.2735	0.5123	0.4026	0.058*	
C10	0.4794 (4)	0.5590 (3)	0.48174 (18)	0.0437 (7)	
H10A	0.5842	0.6014	0.4762	0.052*	
H10B	0.4102	0.6010	0.5195	0.052*	

### Atomic displacement parameters (Å^2^)

**Table:**

	*U*^11^	*U*^22^	*U*^33^	*U*^12^	*U*^13^	*U*^23^
Zn1	0.0373 (2)	0.0380 (2)	0.0379 (2)	−0.00093 (13)	−0.00600 (14)	0.00377 (12)
Cl1	0.0466 (4)	0.0420 (4)	0.0590 (5)	−0.0042 (3)	0.0084 (3)	0.0081 (3)
Cl2	0.0531 (5)	0.0777 (7)	0.0488 (4)	0.0073 (4)	0.0110 (4)	0.0122 (4)
N1	0.0504 (15)	0.0459 (14)	0.0400 (12)	−0.0080 (12)	−0.0086 (10)	0.0022 (11)
N2	0.0651 (19)	0.0498 (16)	0.0520 (15)	−0.0153 (14)	−0.0124 (14)	0.0002 (13)
N3	0.0360 (13)	0.0409 (13)	0.0460 (12)	0.0014 (10)	−0.0065 (10)	0.0084 (11)
N4	0.0400 (13)	0.0421 (14)	0.0422 (12)	0.0022 (11)	−0.0056 (10)	0.0072 (10)
C1	0.073 (2)	0.052 (2)	0.0418 (16)	−0.0170 (17)	−0.0112 (15)	0.0081 (14)
C2	0.073 (2)	0.064 (2)	0.0437 (16)	−0.0237 (19)	0.0012 (16)	0.0009 (16)
C3	0.087 (3)	0.077 (3)	0.0434 (17)	−0.028 (2)	0.0009 (18)	−0.0078 (18)
C4	0.087 (3)	0.051 (2)	0.074 (2)	−0.020 (2)	−0.022 (2)	0.0015 (19)
C5	0.081 (3)	0.057 (2)	0.065 (2)	−0.025 (2)	−0.0131 (19)	−0.0024 (18)
C6	0.0374 (15)	0.0431 (16)	0.0507 (16)	−0.0028 (13)	−0.0089 (12)	0.0069 (13)
C7	0.0449 (17)	0.057 (2)	0.0591 (19)	−0.0017 (15)	0.0001 (14)	0.0220 (16)
C8	0.0363 (16)	0.066 (2)	0.067 (2)	0.0072 (15)	0.0039 (14)	0.0189 (18)
C9	0.0534 (19)	0.0411 (17)	0.0499 (17)	0.0026 (14)	−0.0097 (14)	0.0074 (13)
C10	0.0476 (17)	0.0436 (17)	0.0393 (15)	0.0059 (13)	−0.0007 (12)	0.0060 (12)

### Geometric parameters (Å, °)

**Table:**

Zn1—N3	2.010 (2)	C3—H3A	0.9300
Zn1—N1	2.016 (3)	C4—C5	1.505 (6)
Zn1—Cl2	2.2381 (11)	C4—H4A	0.9700
Zn1—Cl1	2.2567 (9)	C4—H4B	0.9700
N1—C1	1.312 (4)	C5—C5^i^	1.525 (7)
N1—C2	1.363 (4)	C5—H5A	0.9700
N2—C1	1.334 (4)	C5—H5B	0.9700
N2—C3	1.352 (5)	C6—H6A	0.9300
N2—C4	1.472 (5)	C7—C8	1.354 (5)
N3—C6	1.322 (4)	C7—H7A	0.9300
N3—C7	1.371 (4)	C8—H8A	0.9300
N4—C6	1.339 (4)	C9—C10	1.524 (4)
N4—C8	1.380 (4)	C9—H9A	0.9700
N4—C9	1.464 (4)	C9—H9B	0.9700
C1—H1A	0.9300	C10—C10^ii^	1.522 (6)
C2—C3	1.353 (5)	C10—H10A	0.9700
C2—H2A	0.9300	C10—H10B	0.9700
			
N3—Zn1—N1	106.94 (11)	N2—C4—H4B	109.3
N3—Zn1—Cl2	112.43 (8)	C5—C4—H4B	109.3
N1—Zn1—Cl2	110.90 (8)	H4A—C4—H4B	108.0
N3—Zn1—Cl1	106.64 (8)	C4—C5—C5^i^	113.2 (5)
N1—Zn1—Cl1	105.09 (8)	C4—C5—H5A	108.9
Cl2—Zn1—Cl1	114.31 (4)	C5^i^—C5—H5A	108.9
C1—N1—C2	105.3 (3)	C4—C5—H5B	108.9
C1—N1—Zn1	128.8 (2)	C5^i^—C5—H5B	108.9
C2—N1—Zn1	125.7 (2)	H5A—C5—H5B	107.8
C1—N2—C3	107.1 (3)	N3—C6—N4	111.4 (3)
C1—N2—C4	127.4 (3)	N3—C6—H6A	124.3
C3—N2—C4	125.5 (3)	N4—C6—H6A	124.3
C6—N3—C7	105.8 (2)	C8—C7—N3	109.6 (3)
C6—N3—Zn1	126.0 (2)	C8—C7—H7A	125.2
C7—N3—Zn1	127.3 (2)	N3—C7—H7A	125.2
C6—N4—C8	107.1 (3)	C7—C8—N4	106.1 (3)
C6—N4—C9	125.8 (3)	C7—C8—H8A	126.9
C8—N4—C9	127.0 (3)	N4—C8—H8A	126.9
N1—C1—N2	111.7 (3)	N4—C9—C10	113.1 (3)
N1—C1—H1A	124.2	N4—C9—H9A	109.0
N2—C1—H1A	124.2	C10—C9—H9A	109.0
C3—C2—N1	109.4 (3)	N4—C9—H9B	109.0
C3—C2—H2A	125.3	C10—C9—H9B	109.0
N1—C2—H2A	125.3	H9A—C9—H9B	107.8
N2—C3—C2	106.5 (3)	C10^ii^—C10—C9	110.0 (3)
N2—C3—H3A	126.8	C10^ii^—C10—H10A	109.7
C2—C3—H3A	126.8	C9—C10—H10A	109.7
N2—C4—C5	111.5 (3)	C10^ii^—C10—H10B	109.7
N2—C4—H4A	109.3	C9—C10—H10B	109.7
C5—C4—H4A	109.3	H10A—C10—H10B	108.2
			
N3—Zn1—N1—C1	64.7 (3)	C1—N2—C3—C2	1.2 (5)
Cl2—Zn1—N1—C1	−58.2 (3)	C4—N2—C3—C2	179.5 (4)
Cl1—Zn1—N1—C1	177.8 (3)	N1—C2—C3—N2	−1.7 (5)
N3—Zn1—N1—C2	−109.5 (3)	C1—N2—C4—C5	97.3 (5)
Cl2—Zn1—N1—C2	127.6 (3)	C3—N2—C4—C5	−80.7 (5)
Cl1—Zn1—N1—C2	3.6 (3)	N2—C4—C5—C5^i^	176.4 (4)
N1—Zn1—N3—C6	−62.4 (3)	C7—N3—C6—N4	0.4 (4)
Cl2—Zn1—N3—C6	59.6 (3)	Zn1—N3—C6—N4	170.4 (2)
Cl1—Zn1—N3—C6	−174.4 (2)	C8—N4—C6—N3	−0.8 (4)
N1—Zn1—N3—C7	105.5 (3)	C9—N4—C6—N3	−176.6 (3)
Cl2—Zn1—N3—C7	−132.6 (3)	C6—N3—C7—C8	0.1 (4)
Cl1—Zn1—N3—C7	−6.6 (3)	Zn1—N3—C7—C8	−169.7 (2)
C2—N1—C1—N2	−0.7 (4)	N3—C7—C8—N4	−0.6 (4)
Zn1—N1—C1—N2	−175.9 (2)	C6—N4—C8—C7	0.9 (4)
C3—N2—C1—N1	−0.3 (5)	C9—N4—C8—C7	176.6 (3)
C4—N2—C1—N1	−178.6 (4)	C6—N4—C9—C10	−120.0 (3)
C1—N1—C2—C3	1.5 (5)	C8—N4—C9—C10	65.1 (4)
Zn1—N1—C2—C3	176.8 (3)	N4—C9—C10—C10^ii^	−173.2 (3)

Symmetry codes: (i) −*x*−1, −*y*+1, −*z*; (ii) −*x*+1, −*y*+1, −*z*+1.

### Hydrogen-bond geometry (Å, °)

**Table:**

*D*—H···*A*	*D*—H	H···*A*	*D*···*A*	*D*—H···*A*
C3—H3A···Cl2^iii^	0.93	2.77	3.601 (4)	149
C6—H6A···Cl1^iv^	0.93	2.65	3.553 (3)	164

Symmetry codes: (iii) *x*, −*y*+3/2, *z*−1/2; (iv) −*x*, *y*−1/2, −*z*+1/2.

## References

[bb1] Hamada, T., Manabe, K. & Kobayashi, S. (2004). *J. Am. Chem. Soc.* **126**, 7768–7769. 10.1021/ja048607t 15212511

[bb2] Sheldrick, G. M. (1996). *SADABS* University of Göttingen, Germany.

[bb3] Sheldrick, G. M. (2008). *Acta Cryst.* A**64**, 112–122. 10.1107/S0108767307043930 18156677

[bb4] Siemens (1994). *SMART* and *SAINT* Siemens Analytical X-ray Instruments Inc., Madison, Wisconsin, USA.

[bb5] Wang, X. L., Qin, C., Wang, E. B. & Su, Z. M. (2006). *Chem. Eur. J.* **12**, 2680–2691. 10.1002/chem.200501242 16470773

